# A Few Cases Terminating Successfully under the Higher Potencies

**Published:** 1888-06

**Authors:** J. B. Garrison

**Affiliations:** New York


### REPORT OF A FEW CASES TERMINATING SUCCESSFULLY UNDER THE ADMINISTRATION OF THE HIGHER POTENCIES.

J. B. Garrison, M. D., New York.

Mr. President, Gentlemen of the N. Y. S. M. S. I.: In bringing before you a report of some cases which have had a fortunate termination while taking the prescribed remedy in the thirtieth potency or higher, I do not intend to claim that *any particular* potency possesses in itself all warrant of favor, but to give you some of my small experience, and trust it may bring out a discussion beneficial to us all.

In March, 1882, after receiving at the hands of my *Alma Mater* the long-coveted sheepskin, I went to my father's home in the country for a short rest and visit, prior to opening an office for myself.

As in country places generally the advent of a new doctor speedily becomes the subject of gossip, this proved no exception, and I was very soon called upon by neighboring families to prescribe for many minor ailments.

The case which I am about to relate possesses considerable interest, especially from the fact of the drug aggravation made apparent, together with the ultimate relief of all the symptoms.

### Case I.

R. H., sixty, farmer, fully six feet tall; slender and having a decided stoop; temperament, nervous. Health had been poor for many years. Four months previously had a severe attack of what the attending physician, an allopath, had termed neuralgia of the heart, and had controlled the pain entirely by the use of Morphine, hypodermically administered. Was long time recovering—in fact, he has never been quite so well since. Says life has become a burden, as he can get no sleep of a restful nature at all. Sleeps for a short time only, during which he constantly tosses about; then wakens feeling sore and tired all over, and must get up and sit in a chair for a few minutes, then to bed again; and this over and over all night.

Food does not seem to disagree, he says, except salt fish, which he cannot tolerate at all; but he has an all-gone feeling, becoming worse as digestion proceeds, and which is somewhat alleviated after a meal.

The right heart was considerably enlarged, the superior thirds of both lungs, notably the left, were consolidated, and there was a constant tickling at the throat pit, causing a desire to cough, which, however, only resulted in a little white, frothy sputa being raised with a hacking which made his chest more sore. The liver was enlarged downward about two inches, but was not sensitive to the touch.

Shortness of breathing upon the least exertion, together with tumultuous beating of the heart; sensation of constriction across the chest; soreness to the touch of the left subclavicular space.

I had not forgotten the wonderful virtues attributed by Professor Allen, in his lectures on "Materia Medica" to the 200th potency, and I decided here to try it, and, therefore, prescribed the 200th of Phosphorus. I had none, but procured some of Dr. Dunham's preparation from Smith's pharmacy.

My eagerness to cure the case rapidly led me to forget the single dose, and I gave him a package of powders, with directions to take four each day. This was April 4th. He took one powder that afternoon and passed his usual restless night. On the 5th he took one pulv. early in the morning, another at ten A. M., another at three P. M., and lo! instead of improving his condition, as he had hoped, he became worse with each dose, and declared he would take no more until he had seen me.

He could not remain quietly in the house; he was too nervous for that. The weight upon his chest became unbearable when he attempted to walk about, and he panted for breath. It seemed to him as if he must die.

About six P. M. he laid down and slept about half an hour, then took a light tea, and before eight o'clock went to bed, and when he awoke, to his utmost surprise, it was morning, and he had, for the first time in two years, slept all night and awakened feeling rested in the morning.

In the meantime I had been sent for, but being away from home did not see him until the 10th inst., when, upon entering his house, he called out: "Well, Doctor, I am much better than you expected, I guess." He had taken no medicine since the 5th, had slept every night, and was expectorating freely of a yellowish matter, sometimes mixed with blood, and felt much improved in every way. He remarked to me that he ought to have told me that he could not stand strong medicines.

I gave him Sac. Lac., three powders each day, and left one powder of the Phos.$^{cc}$ to be taken on the 12th, which he did with a slight aggravation, which, however, soon passed away.

I saw him again on the 24th, and he was feeling improved in every respect.

He took one more powder of the 200th on the 26th, being all the medicine he received, and on the 15th of May his cough had all ceased, his breathing had become easy, and he had resumed work upon his farm, and he declared himself as well as he had been twenty years ago.

Here was a case in which the medicine unquestionably caused decided aggravation, the patient having no idea what was being given, but felt that each dose was doing him an injury, improvement setting in at once upon stopping the administration of it. He continued perfectly comfortable until one day, after running for about half a mile to meet a coming train, he died suddenly of syncope.

### Case II.

Mr. A. M., ship chandler, æt. forty, presented himself at my office October 29th, 1884, saying he had some soreness about the rectum, which he desired me to attend to. Upon examination I found a complete fistula in ano, the probe entering about half inch to the left of the anus and entering the rectum just below the internal sphincter.

Upon informing him as to the nature of his case, he said he was aware of it, and had been advised by other doctors to submit to an operation, and said he had come to me as he had been told that the homœopaths could cure him with medicine, and that he could not well afford to be away from his business.

I replied that such cures were unquestionably made, and that I would be very glad to try and cure him. He said he felt weak, and there were stitches through the rectum and a constant, slight discharge, and he generally noticed a drop or two of bloody pus upon the sheet in the morning.

Gave him Silicea[illegible]$^{0}$, one dose morning and night, and to report in two weeks. At the end of that time he came and said he felt generally improved, felt stronger, and that the pus was no longer bloody.

Continued same.

In two weeks more he came and reported progress. Once each month thereafter he had his prescription refilled, but I did not see him again until the following March, when he came in one Sunday and asked me if I could not perform an operation for the radical cure of the fistula the next week, as he was tired of waiting for medicine to cure him. I offered to examine him then, but he was in a hurry and could not wait. The next

Sunday he came, and placing him for examination, attempted to pass the probe, but there was, to his great surprise and my infinite delight, no fistula to probe, but only a minute granulating surface which entirely healed in a few days and has remained so ever since.

Did the medicine cure him, or was it a spontaneous cure?

During the whole time of treatment he continued at his business, which was extremely laborious at times, and was often forced to make twenty hours a day's work.

### Case III.

Mr. S., forty years old, Wall Street business man, nervous temperment. Disease: intermittent fever. I had been treating the wife and children of this gentleman for some time, as he considered Homœopathy good enough for women and children, but for himself he wanted medicine. For nearly two years he had had frequent attacks of chills and fever, which were as often suppressed by Quinine, and each time returning with more violence than before.

On the 4th of December, '85, I was surprised by a call to come and see him at once, as he was very ill. To his daughter, who brought the message, I said it would be of little use to see her father, as he would not take my medicine as I should desire him to, but she answered that he was tired of his doctor, and meant to try Homœopathy, as it couldn't do worse than allopathy had for him. As soon as possible I presented myself at his bedside and found him just emerging from a severe chill, which had lasted for about two hours. An old lady who was visiting there had prepared some Acon. in water, and had been giving it every half hour since the chill began. A burning heat succeeded the chill, and that was followed by a profuse perspiration. During the chill he complained of a terrible pain in the stomach, and had much nausea, with vomiting of a large quantity of bile. On account of the large amount of drugs he had taken lately (he had seven different varieties that he was taking every other day) I prescribed, after the paroxysm was over, Nux vom.$^{30}$, a powder every six hours. The next day he felt dull, but not so badly, he said, as after a paroxysm which had been suppressed by Quinine. I said to him then that I should be glad to try to cure him, but I would only consent to make the attempt upon his promise to abide by my treatment, which should be by means of homœopathic medicines alone. He agreed to submit to any treatment I might deem proper. I continued the Nux vom.$^{30}$ every six hours.

December 6th.—Chill came in about half an hour later, but with same symptoms. ℞, Nux.$^{30}$

December 7th.—No chill, but feels dull. Woke at four A. M. No sleep since. ℞, same.

December 8th.—Chill came earlier to-day with the same stomach pains and great yawning and stretching. The heat was the most prominent symptom; was continually thirsty, but wanted only small quantities of water. He begged to have some Quinine, but I refused and prepared some S. L. powders, which he took until the paroxysm subsided, and then gave him Arsen.$^{80}$ every two hours.

December 9th.—This is his off day, and he feels much better.

December 10th.—Chill anticipated but much lighter. ℞, same.

December 11th.—Had a good night's rest, and feels somewhat encouraged.

December 12th.—Chill still anticipated but no pain in the stomach of any account, which greatly pleased him.

December 13th.—Feels pretty fair but dreads to-morrow. ℞, same.

December 14th.—Chill again about same as last. ℞, Ars.$^{30}$

December 15th.—Slept pretty well but feels badly: wonders how soon the chills will cease coming. I wonder too, but I tell him he must be patient.

December 16th.—Chill short, followed by long fever. ℞, Ars.$^{30}$

December 17th.—Has constant desire for stool, but can't accomplish anything. Is very irritable. ℞, Nux.$^{30}$

December 18th.—Chill anticipated three hours but was light; much apprehension; some pain in pit of stomach during the chill. ℞, Nux.$^{30}$

December 19th.—Feels languid, wants to have one dose of Quinine.

December 20th.—Chill again same as last time; chill short, only wants to hover over the fire, heat long; no sweat. Everything has a burning taste. Water seems to generate gas. Eructations of tasteless gas. ℞, Ars.$^{200}$

December 21st.—Feels best of any off day he has had. ℞, Ars.$^{200}$

December 22d.—Chill very light, came one and a half hours later. I now began to see a ray of hope. ℞, Ars.$^{200}$

December 23d.—Appetite much improved, feels better every way. ℞, Ars.$^{200}$

December 24th.—Much annoyed by incarcerated flatulence. Lycop.$^{30}$

December 26th.—Did not see him yesterday, as was out of town, but chill was very slight indeed. ℞, Ars.$^{200}$

December 27th.—Paroxysm scarcely noticeable. ℞, Ars.$^{200}$

December 28th.—Only trouble is the flatulence. Better every other way. Lycop.$^{30}$

December 29th.—Felt in the morning as if chill was coming on, but it did not appear. Felt quite well by evening. Ars.$^{200}$

December 30th.—Found him dressed in his best and on the street, about starting to go to his office, but I persuaded him not to do so. He took a short walk and came back to his home feeling much stronger.

After this he steadily improved without any chills from then up to the present time, and only an occasional dose of the remedy, and now swears by Homœopathy. Here was a case that had been steadily growing worse in spite of Quinine, Iron, Nux vom. in large doses, Fowler's solution of Arsenic, etc., cured by what seems to be no medicine at all, and permanently cured, for he at once went to his office and has continued to work and feel well ever since. Would I have cured him quicker had I given him lower potencies, or did I repeat the higher ones too frequently?

### Case IV.

The last case I shall trouble you to listen to is what might be termed a long-distance cure.

An old Irishwoman, who was in the habit of coming frequently to my office for treatment, said to me one day after I had prescribed for her, that her sister was sick at her house with chills and fever, and wanted me to send her some medicine. I replied that I did not make it a practice to prescribe for unseen patients. She said, "She is too sick to come to-day, and you'd be chargin' double the price if you came to the house, and I can tell you just how she is, and here's the money;" so, on her promise to have her sister come in a day or two, I told her to give me the symptoms.

She was a servant, had lived along the Sound for many years, and had had chills and fever, more or less, for the last dozen years. For two years past had taken Quinine almost every day; for the past week had a severe chill every other day with long heat and no sweat. Feared she was going to die. Restless, always worse after midnight. Very thirsty, but would only take enough water to moisten her lips.

I gave her six powders of Arsen.$^{30}$ and told her to dissolve a powder in six tablespoonfuls of water and give one teaspoonful every two hours.

About three months after this, the old woman again presented herself at my office, and I asked her why her sister never came as she promised. "Ah! Doctor," said she, "those powders was the great stuff indeed; she took them and has never had another chill since, and is working as well as ever, and don't need any doctor at all."

